# Patient involvement in resident assessment within the Competence by Design context: a mixed-methods study

**Published:** 2019-03-13

**Authors:** Katherine Moreau, Kaylee Eady, Mona Jabbour

**Affiliations:** 1 Faculty of Education, University of Ottawa, Ontario, Canada; 2 Department of Emergency Medicine, Faculty of Medicine, University of Ottawa, Ontario, Canada; 3 Department of Pediatrics, Faculty of Medicine, University of Ottawa, Ontario, Canada; 4 Children’s Hospital of Eastern Ontario, Ontario, Canada

## Abstract

**Background:**

Patients can contribute to resident assessment in Competence by Design (CBD). This study explored the extent, nature, as well as the facilitators and hindrances of patient involvement in resident assessment within and across Canadian specialty/sub-specialty/special programs that are transitioning or have transitioned to CBD.

**Methods:**

We used a two-phase sequential explanatory mixed-methods design. In Phase 1, we surveyed program directors (PDs). In Phase 2, we interviewed PDs from Phase 1.

**Results:**

In Phase 1, 63 (62.4%) respondents in the CBD preparation stage, do not know if patients will be involved in resident assessment, 21 (20.8%) will involve patients, and 17 (16.8%) will not involve patients. Of those in the field-testing or implementation stages, 24 (72.7%) do not involve patients in resident assessment, five (15.2%) do involve patients, and four (12.1%) do not know if they involve patients. In Phase 2, 12 interviewees raised nine factors that facilitate or hinder patient involvement including, patients’ interests/abilities, guidelines/processes for patient involvement, type of Entrustable Professional Activities, type of patient interactions in programs, and support from healthcare organizations.

**Conclusion:**

Patient involvement in resident assessment is limited. We need to engage in discussions on how to support such involvement within CBD.

## Introduction

Competency-based medical education (CBME) requires multiple assessors of residents,^1^^,^^2^ authentic assessment, and multi-faceted assessment programs.^1^^,^^3^ Given the emphasis that CBME places on assessment *for* learning as well as the range of competencies included in, for example, the CanMEDS framework, it is essential to develop assessment programs that encompass a range of assessment strategies and assessors.^1^ Patients and family members or caregivers (herein referred to as patients), if provided with the opportunity, have the ability to contribute to these assessment programs. Residency programs can include assessments by patients as part of multisource feedback (MSF).^4^ Used for the assessment of competencies, MSF can include the collection of data on residents’ performance from peers, supervising physicians, allied health professionals, and patients. By including patient assessments in MSF, residency programs can ensure that patients’ first-hand experiences with residents as well as their perceptions of residents’ skills and abilities are included as part of the learning process.

Researchers suggest that residents need assessments from patients to improve their performance,^5^ to develop and maintain patient-centred professionalism,^6^ to develop positive physician-patient relationships,^7^ and to understand how their interactions with patients impact health outcomes.^8^ Residents have reported high satisfaction with and utilization of patient assessments.^9^^,^^10^ Patient involvement in resident assessment can also empower patients to improve the care that they and others receive.^11^^,^^12^ In recognition of these mutual benefits, the Royal College of Physicians and Surgeons of Canada (RCPSC) recommends the involvement of patients in resident assessment in their CBME model, entitled Competence by Design (CBD). However, there is limited research on patient involvement in resident assessment.^13^ Towle and Godolphin’s bibliography on patient involvement in health professions education noted that a mere 29 of 657 (4.4%) studies focused on patient involvement in assessment, seven of which are from medicine.^14^ These studies found that patients can assess residents’ professionalism, collaboration, interpersonal abilities, and communication skills.^7^^,^^15^^-^^17^ Authors also showed that patients can provide reliable assessments of residents’ skills.^12^^,^^18^^,^^19^ Nonetheless, these studies did not explore the extent and nature of patient involvement in resident assessment within the emerging Canadian CBD context. Thus, the purpose of this two-phase sequential explanatory mixed-methods study was to explore and document the current state of and plans for patient involvement in resident assessment within these CBD programs to identify resources and activities needed to advance and sustain this important component of resident assessment. The following research questions, as they related to CBD, guided our study:

### Phase 1: Survey

1. To what extent are patients or will patients be involved in resident assessment?
2. Why are patients or will patients be involved or not involved in resident assessment?
3. What skills and abilities can patients assess?

### Phase 2: Interviews

1. What factor(s) facilitate and hinder patient involvement in resident assessment?

## Methods

### Study design

We used a two-phase sequential explanatory mixed-methods design.^20^ Phase 1 encompassed quantitative survey data from program directors (PDs) whose programs were in the CBD preparation, field-testing, or implementation stages at the time of the study. We then used the findings from Phase 1 to inform participant-level questions and identify participants for Phase 2, which comprised qualitative interview data with selected PDs. Our rationale for using interviews was pragmatic. When designing the study, we recognized that it would be challenging to schedule focus groups with busy PDs but that one-on-one interviews would allow the PDs to participate at times that were most convenient for them. Nevertheless, the quantitative and qualitative study portions were equally important in terms of addressing the research topic.^20^ We opted for this mixed methods design because we anticipated that the qualitative interview findings (Phase 2) would build on the initial quantitative survey results (Phase 1) and thus, provide a more comprehensive understanding of patient involvement in resident assessment than either quantitative or qualitative designs alone.^20^ This design also enabled us to create and ask specific interview questions to PDs who responded “yes,” “no,” or “don’t know” to questions of patient involvement in resident assessment on their Phase 1 survey and thereby elicit and illustrate accurate descriptions and understandings of the factor(s) that facilitate and hinder patient involvement in resident assessment. We obtained ethics approval for both Phases 1 and 2 from the University of Ottawa (File number 03-17-08).

### Phase 1: Survey

#### Sample

Based on the RCPSC’s website, we identified 17 primary specialties, 12 sub-specialties, and one special program (i.e., Surgical Foundations) that were in the CBD preparation, field-testing, or implementation stages. We distributed the online survey to 462 program directors (PDs).

#### Instrument development

We developed survey dimensions and items using literature on strategies for investigating patient involvement in medical education^9^^,^^13^^,^^21^^,^^22^ and assessment practices.^16^^,^^23^^-^^25^ Survey items pertained to one of the following dimensions: (a) level and type of patient involvement; (b) purpose of assessment; (c) reasons for patient involvement; and (d) skills and abilities. Six reviewers knowledgeable in patient involvement in medical education, assessment, and CBD reviewed the survey. We also piloted it with five former PDs and five physician-educators ineligible for the study. We then translated the 23-item survey to French and piloted it with three former French PDs.

#### Data collection procedures

A Research Assistant (RA) emailed the survey link to PDs. We confidentially tracked responders/non-responders and used a modified version of Dillman’s et al.’s Tailored Design Method.^26^

#### Data analysis

We calculated descriptive statistics (i.e., frequencies, percentages) in SPSS (version 24).

### Phase 2: Interviews

#### Sample

We used purposeful criterion-based sampling to identify and recruit PDs. To be eligible for an interview, PDs had to have completed the Phase 1 survey and expressed interest in participating in a Phase 2 interview.^27^

#### Instrument development

We used Phase 1 findings to inform the semi-structured interview guides. We developed separate guides for those who responded “yes,” “no,” or “don’t know” in regard to patient involvement in resident assessment in Phase 1. Each guide consisted of an introductory script and six open-ended questions on resources, values, or conditions that affect programs’ abilities to involve patients in resident assessment. We piloted the guides with five former English-speaking PDs, translated them to French, and then piloted them with three former French-speaking PDs.

#### Data collection procedures

The RA emailed information letters to eligible PDs, requesting them to reply if they were interested in participating. Each interview lasted 30 to 60 minutes. All interviewees provided informed consent prior to the interview. The RA conducted each interview by telephone, audio-recorded it, and transcribed it verbatim.

#### Data analysis

Two of us (KM & KE) analyzed the data ourselves and recognized that our interpretations of the data were mediated through us as medical education researchers and proponents of patient involvement in medical education and CBD. Miles’ and Huberman's three-step iterative process (i.e., data condensation, data displays, drawing and verifying conclusions)^28^ informed our inductive analyses. The goal of the analyses was to identify the major themes articulated by the PDs representing factors that facilitate or hinder patient involvement in resident assessment. Throughout the analyses, we identified factors that were present in more than one interview.

Following each interview, we independently listened to the audio-recordings to identify factors that interviewees highlighted as facilitators or hindrances to patient involvement in resident assessment. We documented these factors to establish an audit trail. Following transcription, the RA compared the transcripts to the audio-recordings. We then independently reviewed the transcripts and summaries to create our own coding systems. We read each transcript, annotated phrases, and coded the data. We incorporated codes not previously identified in our listening to the audio-recordings. We then met to discuss our independent analyses. Together, we merged our analyses, created data display tables, and generated thematic conclusions, including exemplar quotations.

## Results

### Phase 1: Survey

#### Demographics

We obtained 134 completed surveys (response rate of 29.0%). When asked about their programs’ CBD stage, 101 (75.4%) indicated CBD preparation (i.e., the specialty committee is working with the RCPSC to prepare the program for CBD), 21 (15.7%) selected CBD field-testing (i.e., the program is field testing aspects of CBD), and 12 (9.0%) stated CBD implementation (i.e., residents are in a CBD-based program). Tables 1 and 2 provide demographic details.

**Table 1:** Survey respondents’ demographic characteristics (N = 134)

Characteristic	n (%)
**Gender**	
Female	61 (45.5)
Male	64 (47.8)
Prefer not to specify	9 (6.7)
**Years working as a Program Director**	
<12 months	32 (23.9)
1-5 years	65 (48.5)
6-10 years	25 (18.7)
11-15 years	4 (3.0)
>20 years	1 (0.7)
Prefer not to specify	7 (5.2)
**Region of Canada**	
British Columbia	12 (9.0)
Alberta	14 (10.4)
Saskatchewan	6 (4.5)
Manitoba	7 (5.2)
Ontario	38 (28.4)
Québec	36 (26.9)
Nova Scotia	6 (4.5)
Newfoundland and Labrador	1 (0.7)
Prefer not to specify	14 (10.4)

**Table 2:** Survey respondents’ programs (N = 134)

Program	n (%)
Anesthesiology	4 (3.0)
Cardiology	10 (7.5)
Critical Care Medicine	8 (6.0)
Emergency Medicine	4 (3.0)
Gastroenterology	2 (1.5)
General Internal Medicine	7 (5.2)
Geriatric Medicine	2 (1.5)
Internal Medicine	6 (4.5)
Medical Oncology	5 (3.7)
Neonatal-Perinatal Medicine	2 (1.5)
Nephrology	6 (4.5)
Pediatrics	12 (9.0)
Physical Medicine & Rehabilitation	3 (2.2)
Psychiatry	5 (3.7)
Radiation Oncology	6 (4.5)
Respirology	4 (3.0)
Rheumatology	6 (4.5)
Cardiac Surgery	2 (1.5)
General Surgery	5 (3.7)
Neurosurgery	3 (2.2)
Obstetrics & Gynecology	7 (5.2)
Otolaryngology	6 (4.5)
Plastic Surgery	2 (1.5)
Surgical Foundations	3 (2.2)
Urology	3 (2.2)
Anatomical Pathology	2 (1.5)
Clinical Immunology & Allergy	3 (2.2)
Forensic Pathology	1 (0.7)
General Pathology	1 (0.7)
Nuclear Medicine	4 (3.0)

#### To what extent are patients or will patients be involved in resident assessment?

Of the 101 respondents in the CBD preparation stage, 63 (62.4%) stated that they did not know if patients will be involved in resident assessment, 21 (20.8%) said that they will involve patients, and 17 (16.8%) indicated they will not involve patients. Among the 33 respondents in the field-testing or implementation stages, 24 (72.7%) said that they do not involve patients in resident assessment, five (15.2%) stated that they do involve patients, and four (12.1%) stated that they do not know if they involve patients.

#### Preparation stage.

Ten (47.6%) of the 21 respondents who are in the preparation stage and who indicated that they will be involving patients in resident assessment stated that they did not know how often patients will be involved over a four-week period. The remaining respondents stated that patients will never (n=2, 9.5%), rarely (n=4; 19.0%), sometimes (n=4; 19.0%), or frequently (n=1; 4.8%) be involved over a four-week period. Twelve (57.1%) of these 21 respondents stated that patients will be involved in formative assessment, one (4.8%) stated that patients will be involved in summative assessment, and eight (38.1%) responded that they did not know if patients will be involved in formative or summative assessment. When asked if patients will provide written assessments, ten of the 21 respondents (47.6%) noted that they did not know, six (28.6%) indicated that patients will provide written assessments, and five (19.0%) said that patients will not. In regard to verbal assessments, over half (n=11; 52.4%) of the 21 respondents said “yes” patients will provide verbal assessments, seven (33.3%) said they “did not know,” and three (14.3%) said “no.” Four (19.0%) of the 21 answered that patients will assess residents in Objective Structured Clinical Examinations (OSCEs) and all confirmed that patients will not assess residents in simulation. All 21 respondents indicated that patients will not be involved in assessment tool development.

#### Field-testing or implementation stage.

Of the five respondents in the field-testing or implementation stages who stated that they involve patients in resident assessment, four (80.0%) stated that patients are rarely involved in the assessment of residents over a four-week period and one (20%) indicated that they are frequently involved. All five stated that patients are involved in formative rather than summative assessment. In terms of providing written assessments, three (60.0%) indicated that patients provide written assessments. When asked about verbal assessments, two (40.0%) said patients provide them. All respondents confirmed that patients are not involved in assessments for OSCEs or simulation. They also noted that patients are not involved in assessment tool development.

#### Why are patients or will patients be involved or not involved in resident assessment?

The main reason why respondents in the preparation and field-testing or implementation stages will be involving or do involve patients in resident assessment is to provide and gain firsthand information on the care provided by residents. This reason was cited by 16 (76.2%) of the 21 programs in the preparation stage and four (80.0%) of the five programs in the field-testing or implementation stages that will be involving patients in assessment. Other reasons indicated by respondents included: (a) to improve the quality of care (57.1% preparation stage, 40.0% field-testing or implementation stages); (b) to empower patients (28.6% preparation stage, 20.0% field-testing or implementation stages); and (c) to satisfy program accreditation requirements (19.0% preparation stage, 40.0% field-testing or implementation stages). Appendix A provides a list of reasons why patients might not be or are not involved in resident assessment.

#### What skills and abilities can patients assess?

Table 3 presents respondents’ views on which skills and abilities patients can (and cannot) assess. Across the CBD stages and irrespective of patient involvement in resident assessment, the majority of respondents thought that patients could assess residents’ communication and respectfulness.

**Table 3:** Skills and abilities patients can assess

Patient Involvement	Skills and Abilities	Preparation Stage N =	Field-testing or Implementation Stages
		n (%)	
Yes		**N= 21**	**N=5**
	Communication	20 (95.2)	4 (80.0)
	Team work	8 (38.1)	2 (40.0)
	Leadership	4 (19.0)	1 (20.0)
	Situational awareness (“Knowing what is going on around you”)	8 (38.1)	1 (20.0)
	Decision making	4 (19.0)	0 (0.0)
	Coping with stress	5 (23.8)	0 (0.0)
	Coping with fatigue	1 (4.8)	0 (0.0)
	Respectfulness	18 (85.7)	4 (80.0)
	Punctuality	13 (61.9)	1 (20.0)
	Awareness of limitations	6 (28.6)	1 (20.0)
	Ability to ask for help	10 (47.6)	1 (20.0)
	Comfort level in a clinical setting	12 (57.1)	3 (60.0)
	Adaptability	4 (19.0)	1 (20.0)
	Managing workloads	1 (4.8)	1 (20.0)
	Resolving conflicts	8 (38.1)	1 (20.0)
	Patients cannot assess residents	0 (0.0)	0 (0.0)
Don’t Know		**N=63**	**N=4**
	Communication	58 (92.1)	4 (100.0)
	Team work	21 (33.3)	1 (25.0)
	Leadership	12 (19.0)	1 (25.0)
	Situational awareness (“Knowing what is going on around you”)	28 (44.4)	3 (75.0)
	Decision making	16 (25.4)	2 (50.0)
	Coping with stress	11 (17.5)	3 (75.0)
	Coping with fatigue	2 (3.2)	1 (25.0)
	Respectfulness	56 (88.9)	3 (75.0)
	Punctuality	39 (61.9)	3 (75.0)
	Awareness of limitations	19 (30.2)	3 (75.0)
	Ability to ask for help	17 (27.0)	2 (50.0)
	Comfort level in a clinical setting	30 (47.6)	2 (50.0)
	Adaptability	14 (22.2)	2 (50.0)
	Managing workloads	3 (4.8)	2 (50.0)
	Resolving conflicts	19 (30.2)	3 (75.0)
	Patients cannot assess residents	0 (0.0)	0 (0.0)
No		**N=17**	**N=24**
	Communication	14 (82.4)	20 (83.3)
	Team work	3 (17.6)	5 (20.8)
	Leadership	2 (11.8)	2 (8.3)
	Situational awareness (“Knowing what is going on around you”)	5 (29.4)	10 (41.7)
	Decision making	3 (17.6)	6 (25.0)
	Coping with stress	6 (35.3)	7 (29.2)
	Coping with fatigue	5 (29.4)	3 (12.5)
	Respectfulness	13 (76.5)	20 (83.3)
	Punctuality	11 (64.7)	14 (58.3)
	Awareness of limitations	5 (29.4)	7 (29.2)
	Ability to ask for help	2 (11.8)	9 (37.5)
	Comfort level in a clinical setting	8 (64.7)	13 (54.2)
	Adaptability	2 (11.8)	7 (29.2)
	Managing workloads	2 (11.8)	3 (12.5)
	Resolving conflicts	4 (23.5)	7 (29.2)
	Patients cannot assess residents	1 (5.9)	1 (4.2)

### Phase 2: Interviews

#### Demographics

We interviewed 12 PDs; eight (66.7%) in the CBD preparation stage and four (33.3%) in the field-testing or implementation stages. Of the interviewees in the preparation stage, four (50.0%) reported that patients will be involved in resident assessment and four (50.0%) said that they did not know if patients will be involved. All interviewees in the field-testing or implementation stages noted that patients are not involved in resident assessment. The interviewees represented Anesthesiology, General Surgery, Medical Oncology, Neonatal-Perinatal Medicine, Nephrology, Obstetrics and Gynecology, Otolaryngology, Pediatrics, Respirology, and Surgical Foundations from various geographical locations.

#### What factor(s) facilitate or hinder patient involvement in resident assessment?

Factors that some interviewees viewed as facilitators others instead viewed as hindrances (see Table 4). Appendix B provides exemplar participant quotations.

**Table 4:** Factors that facilitate and hinder patient involvement in resident assessment by stage of CBD

Factor	Preparation stage	Field-testing or implementation stages
Patients’ interests and abilities.	–	–
Funding	–	–
Guidelines and processes for patient involvement in assessment	–	–
Faculty members’ and residents’ perceptions	+ –	–
Staffing and time	+ –	–
Availability and existence of patient assessment tools	+ –	–
Type of Entrustable Professional Activities	+ –	–
Type of patient interactions in program	+ –	–
Support from healthcare organizations	+ –	–

*Note*.**–** indicates that the interviewees perceived the factor to hinder patient involvement in resident assessment; **+ –** indicates that the interviewees perceived the factor to both hinder and facilitate patient involvement in resident assessment

#### Patients’ interests and abilities

All participants in the preparation and field-testing or implementation stages, irrespective of their desires to involve patients in assessment, thought that patients’ interests and abilities to be involved will be/is a hindrance. They commented on how they believe that patients will/do not want to be involved in education or that they will/do not have the time to participate in assessment. Interviewees also expressed concern about the expertise of patients to assess residents. They noted that patients would need training in resident assessment and medical education requirements. Interviewees thought that providing such training is unfeasible given current program resources.

#### Funding

Regardless of the participants’ CBD stage and their interest in involving patients in resident assessment, they all said that insufficient funding hinders them from involving patients. Participants commented on how they would require additional funding to implement and ensure ongoing patient involvement. They discussed how patient involvement is costly and impossible within existing budgets.

#### Guidelines and processes for patient involvement in assessment

Participants across all CBD stages viewed the lack of guidelines and processes as a hindrance to patient involvement in resident assessment. They noted the non-existence of guidelines and processes on how to collect resident assessment data from patients, which patients they should involve, how many patient assessments they should collect, what patient assessment reports should include, and if these reports should only include anonymized data. Several interviewees also commented on how the online assessment system will be/is restricted to faculty members and that there are no guidelines or processes on how patients will/can access it. They expressed that guidelines and processes would facilitate and standardize patient assessment activities but that without them, patient assessments will be/are haphazard or not occur. Moreover, most participants noted that they would not involve patients in resident assessment unless it was mandatory.

#### Faculty members’ and residents’ perceptions

Some interviewees in the preparation stage described how their positive perceptions towards patient involvement in resident assessment would facilitate their efforts to involve patients. These participants believe that patients will bring unique insights to well-rounded assessment programs and provide residents with important information that they can use to improve their competencies. However, others in the preparation, field-testing, or implementing stages flagged that residents’ and faculty members’ prior preconceived negative perceptions towards patient assessments might hinder involvement. They commented on how they may not take it seriously. Participants also discussed how they perceive patient assessments to be biased (i.e., overly positive or negative) and thus, hinders them from collecting and using them. They noted that if they, as the PDs, are not supportive of patient assessments then they will not occur, as they champion the various assessment strategies. Furthermore, several of these interviewees disclosed that they do not see patient assessments as a priority.

#### Staffing and time

Participants discussed staffing and time as factors that both facilitate and hinder patient involvement in resident assessment. They focused on the amount of time available for staff to collect patient assessments and the willingness of staff and residents to assume such responsibilities. Some interviewees in the preparation stage explained how they would ask clerks to distribute assessment forms to patients. They also suggested that nurses could assist or residents could collect the patient assessments. However, others within the preparation stage detailed how they will not have the staff or time to collect patient assessments. They discussed how the time commitments of teaching and clinical responsibilities would hinder them from involving patients in resident assessment. Those in the field-testing or implementation stages echoed these thoughts and added that they do not have anyone in their programs who can assume responsibility for overseeing and collecting patient assessments. They also explained that residents would forget to collect these assessments and thus, it would be impractical to assign the task to them.

#### Existence and availability of patient assessment tools

Participants whose programs are in the preparation stage thought that the existence and availability of patient assessment tools will be both a facilitator and hindrance to patient involvement in assessment. As a facilitator, some explicated how they plan to involve patients in resident assessment using existing tools within their 360° assessment systems. However, others in the preparation stage noted that they do not have access to or are unaware of existing patient assessment tools, especially those with validity and reliability evidence. Interviewees in the field-testing or implementation stages also reiterated their lack of access to and knowledge of patient assessment tools as a hindrance. They noted the need to develop patient-specific assessment tools that align with their existing Entrustable Professional Activities (EPAs), but they explained that the development of such tools is beyond their expertise.

#### Type of Entrustable Professional Activities

Some participants in the preparation stage highlighted how the EPAs that they are developing for their programs will require or are amenable to patient involvement in resident assessment. In some cases, they described how it would be essential to obtain patient assessments to know if residents are progressing appropriately. Conversely, others in the preparation, field-testing, or implementation stages stressed how their programs’ EPAs will hinder their abilities to involve patients because the EPAs are not patient-oriented. They stated that the EPAs for their programs do not require patient assessments. They explained how the EPAs focus solely on skills/abilities that only require faculty members’ assessments. These interviewees also noted that they will/have not included patients as assessment sources and thus, patients will/are not involved.

#### Type of patient interactions in program

Several participants in the preparation stage discussed longitudinal patient interactions in their programs. They believed that these interactions would facilitate their abilities to incorporate patient assessments and enable patients to comment on residents’ competencies over time. However, other interviewees in the preparation, field-testing, or implementation stages commented on how their types of patient interactions are not conducive to patient involvement in resident assessment. They explained how interactions are episodic or short and therefore, patients will/do not have enough time with residents to provide reliable and constructive assessments.

#### Support from healthcare organizations

****.**Participants in the preparation stage who plan to involve patients explained how support from the academic hospitals in which their residents are training would be essential for facilitating patient involvement in resident assessment. They noted that they are involving administrators as well as patient advisory groups in preparation efforts to understand how they can best involve patients. These interviewees detailed how these stakeholders will facilitate their programs’ efforts to contact patients for assessment activities. They discussed how these stakeholders are also providing them with advice on how to respect patients’ privacy concerns when involved in resident assessment. Participants mentioned that the patient-centred philosophies of their academic hospitals would support patient involvement in resident assessment. Nonetheless, those in the field-testing and implementation stages said regardless of their hospitals’ philosophies, they do not have staff or mechanisms to assist and guide them in involving patients in resident assessment.

## Discussion

This study provides an understanding of the current extent, nature, as well as the facilitators and hindrances of patient involvement in resident assessment. The majority of Phase 1 respondents in the CBD preparation stage indicated that they did not know if patients would be involved in resident assessment. This uncertainty is not surprising as these programs are still in early CBD stages and navigating the transition process. However, the majority of respondents in the field-testing or implementation stages indicated that they do not involve patients in resident assessment. This finding does not bode well for patient involvement in resident assessment, as many of the programs in the preparation stage may look to these early CBD adopters, who are piloting the model to ensure that it is appropriate and feasible before implementing it across all programs, to inform their programs’ assessment activities.^29^ This lack of patient involvement in assessment also differs from the methods used to assess residents in Accreditation Council for Graduate Medical Education (ACGME) programs in the United States.^24^ Holt et al. in their review of methods used to assess residents in ACGME programs found that 61% of programs involved patients as assessors and that 34% included family members as assessors.^24^

All respondents in the present study also noted a lack of patient involvement in the development of assessment tools. This finding is unfortunate as research shows that patients can contribute to tool creation.^30^^-^^32^ Moreover, participants in Phase 2 commented on having no access to or awareness of patient assessment tools. However, there are several existing patient assessment tools with validity and reliability evidence that programs could use as part of their MSF activities.^31^^,^^33^^-^^37^ Nevertheless, researchers and other RCPSC stakeholders could better promote these tools to PDs to facilitate use. In addition, prior to using any existing tools, programs need to ensure that the tools’ items align with their programs’ EPAs, as these EPAs should be used as the blueprints that guide tool selection.^38^^,^^39^

On another note, participants in the current study who will or do involve patients in resident assessment noted how patient involvement provides patients’ perspectives to residents, improves care provision, and empowers patients. These benefits are consistent with the literature on patient involvement in assessment.^7^^,^^40^^-^^42^ That said, some participants discussed how they will or are only involving patients in resident assessment to satisfy program accreditation requirements. Unfortunately, this form of involvement often leads to token patient involvement.^8^

Phase 1 participants identified that patients could best assess residents’ communication and respectfulness, which is congruent with previous studies.^7^^,^^16^^,^^24^ In relation to the assessment of these skills, interviewees also noted that opportunities for longitudinal patient-resident interactions would facilitate patient involvement. Researchers have shown that a series of longitudinal patient assessments can illustrate learners’ evolution of patient-oriented behaviours and communication abilities.^43^ Conversely, other interviewees detailed how resident-patient interactions in their programs are episodic or short and thus not conducive to patient involvement in resident assessment. However, as we have shown in a previous study, parent assessments of residents is feasible in pediatric emergency departments, where interactions are brief and isolated.^31^

In both Phases 1 and 2, regardless of CBD stage or intentions of involving patients, participants indicated several reasons why they might not or do not involve patients in resident assessment, including lack of funding and time. Holmboe et al.^38^ and Pinsk et al.^44^ confirm that CBME and CBD, respectively, lead to increased demands on faculty and resources and that assessment efforts are time-intensive. Thus, adding patient assessments is undoubtedly challenging. Participants also suggested that their lack of knowledge on how to involve patients in resident assessment is a reflection of a dearth of guidelines and processes on how to do this. Such guidelines and processes can support a culture of patient inclusion^8^^,^^12^ and include information on remuneration for patient assessors,^17^ strategies for increasing the diversity of patients involved in assessment,^45^ and data collection techniques.^32^

Of interest, Phase 2 interviewees highlighted hindrances and facilitators to involving patients in resident assessment that were not strongly represented in Phase 1. For example, while a small number of Phase 1 respondents indicated that their programs do not believe patients can assess residents, all Phase 2 participants thought that patients’ interests and abilities will be or are a hindrance to patient involvement in resident assessment. Interviewees focused heavily on patients’ lack of expertise to assess residents. Other researchers have commonly cited concerns about overburdening patients or about patients’ abilities to provide meaningful and reliable assessments because of their emotional, physical, or mental health.^46^ Few have commented specifically on patients’ expertise. Those that have alluded to patients’ expertise suggested that faculty members may feel threatened by the transfer of some assessment power from themselves to patients and thus, resist it,^47^ or commented that programs have provided minimal or inappropriate training to patients to enable effective assessment involvement.^48^

Interviewees also expanded on staffing as a factor that can both hinder and facilitate patient involvement in resident assessment. Some noted that their programs will not or do not have the staff to collect patient assessments. Naylor et al.^17^ echoed these participants’ concerns noting that the Canadian context has deficiencies in infrastructure for supporting authentic patient involvement. However, other interviewees also commented on the potential of having other health professionals and residents assist with patient assessments. Previous research studies^35^^,^^36^^,^^49^ have successfully used such processes, but it is unclear how these processes would work in Canadian healthcare systems where health professionals’ job descriptions do not include the collection of patient assessments.

Lastly, interviewees raised the importance of having leadership and support to facilitate patient involvement from both the healthcare organizations in which the residents provide care and the PDs themselves. A lack of sustained leadership and support is one of the main reasons why patient involvement in medical education is not mainstream.^45^ In order to facilitate such involvement, Towle et al.^45^ endorse the promotion of patient involvement “through directives such as accreditation standards, external and internal policies, pronouncements from professional bodies and best practice statements.”

### Limitations & Future Directions for Research

This study has four limitations that future research can mitigate. First, some may view the survey response rate of 29.0% as a limitation. While it is plausible that the data would have yielded different results if the response rate was higher, the response rate is consistent with other surveys targeted at PDs^50^ and those involving healthcare professionals.^51^ However, future research in this area could work on increasing this response rate. Second, since we limited the survey to the perspectives of PDs for specialty/sub-specialty/special programs associated with the RCPSC, in future, it would be interesting to survey PDs for Family Medicine programs. Third, many respondents from Phase 1 elected to not participate in Phase 2. We do not know if there were any differences between those who participated in Phase 2 and those who did not. It is possible that PDs who participated where more interested in the topic and thus, expressed different views from those who did not participate. Lastly, although Phase 2 included a small group of PDs, it included the views of PDs from several specialty/sub-specialty/special programs and various geographical locations. Nevertheless, it would be beneficial to undertake future studies to gather additional PDs’ perspectives on the topic. Moreover, patients and residents are key stakeholders on this topic, and future studies should also explore their views. Such studies could use focus groups, rather than one-on-one interviews, in order to facilitate the obtainment of high-quality data, as the interactional, synergistic nature would encourage these stakeholders to clarify or expand upon their discussion points in relation to those raised by others.

Patient involvement in resident assessment appears limited and sporadic across Canadian specialty, sub-specialty, and special programs that are transitioning or have transitioned to CBD. Unfortunately, the majority of respondents whose programs are in the CBD field-testing or implementation stages indicated that they do not involve patients in resident assessment. This lack of involvement will inevitably have a deleterious impact on the extent of patient involvement in resident assessment, since other programs may follow the examples and activities of these early CBD adopters. The PDs also identified factors that facilitate and hinder such involvement. Overall, by highlighting the current state of patient involvement in resident assessment as well as these factors, we are optimistic that those leading CBD transitions will be motivated to engage in critical discussions about the extent to which, how, and why we need to enact measures to better support patient involvement in resident assessment. After all, since patients are central to healthcare and medical education, residents may benefit from and appreciate the involvement of patients in their assessment.

## Appendix A

**Table A1:** Why patients might not be or are not involved in resident assessment

Patient Involvement	Why patients might not be or are not involved in resident assessment	Preparation Stage	Field-testing or Implementation Stages
		n (%)	
Yes		**N=21**	**N=5**
	Residents do not have direct contact with patients	2 (9.5)	0 (0.0)
	Program doesn’t know how to involve patients in resident assessment	10 (47.6)	2 (40.0)
	No funding to support patient involvement in resident assessment	9 (42.9)	1 (20.0)
	No time to support patient involvement in resident assessment	8 (38.1)	1 (20.0)
	No tools to support patient involvement in resident assessment	12 (57.1)	3 (60.0)
	Program does not believe patients can assess residents	1 (4.8)	3 (60.0)
	Patients’ health conditions impede them from assessing residents	6 (28.6)	1 (20.0)
	Don’t know	1 (4.8)	1 (20.0)
Don’t Know		**N=63**	**N=4**
	Residents do not have direct contact with patients	1 (3.2)	0 (0.0)
	Program doesn’t know how to involve patients in resident assessment	30 (47.6)	1 (25.0)
	No funding to support patient involvement in resident assessment	35 (55.6)	2 (50.0)
	No time to support patient involvement in resident assessment	35 (55.6)	1 (25.0)
	No tools to support patient involvement in resident assessment	43 (68.3)	2 (50.0)
	Program does not believe patients can assess residents	3 (4.8)	0 (0.0)
	Patients’ health conditions impede them from assessing residents	14 (22.2)	2 (50.0)
	Don’t know	2 (3.2)	0 (0.0)
No		**N=17**	**N=24**
	Residents do not have direct contact with patients	2 (11.8)	0 (0.0)
	Program doesn’t know how to involve patients in resident assessment	5 (29.4)	13 (54.2)
	No funding to support patient involvement in resident assessment	3 (17.6)	14 (58.3)
	No time to support patient involvement in resident assessment	4 (23.5)	13 (54;2)
	No tools to support patient involvement in resident assessment	7 (41.2)	15 (62.5)
	Program does not believe patients can assess residents	3 (17.6)	1 (4.2)
	Patients’ health conditions impede them from assessing residents	3 (17.6)	1 (4.2)
	Don’t know	1 (5.9)	0 (0.0)

## Appendix B

**Table B1:** (Supplementary material) Exemplar quotations for factors that facilitate and hinder patient involvement in resident assessment by stage of CBD

Factor	Quotations	
	Preparation stage	Field-testing or implementation stages
**Patients’ interests and abilities**		
As a facilitator	NA	NA
As a hindrance	Patients are not in the role to educate the residents (DK02). It’s not appropriate to ask them for feedback, they are stressed and don’t know how (DK03). How would you educate them? Do you know what I mean, in kind of the lingo, the confidentiality aspects, you know, all those things I think are things we need to think about (Y03). Patients need education, you know, what is a resident, what is a first year resident, what is a first year resident expected, what is a fourth year resident expected to do (Y04)? I question how the average run of the mill individual that’s seeking medical care is going to be capable of determining what they think is appropriate [in regards to resident competency]….they have no concept of what medical training is (DK01).	Don’t even qualify to assess a doctor or resident (N01). In a clinical encounter often we’re running late whenever you have residents in your clinic and the patient tries to leave as soon as they can when they’re done right….won’t want to stay to assess (N02). Should we ask our patients admitted on the ward? But they are some of the sickest patients. Are they really going to feel like completing an assessment…? So…I just don’t know (N03).
**Funding**		
As a facilitator	NA	NA
As a hindrance	It’s very expensive…it would be a massive undertaking…think about how that’s going to financially pan out…they’re not supplying any of the money that’s going to get my faculty interested….I suspect that unless there is some significant investment in some way this is going to kind of fall off the map (DK01). Funding is something that is required to be able to do this…I don’t necessarily think we have it (Y03). Funding is a big thing…big part of the problem (Y04).	The funding is going to be the same, all the onus is on…is on me [Program Director] to reorganize everything without any additional resources (N01). It’s too hard to figure out the logistics and the funding (N02). It’s resources! They give us about 25 cents during the year to run the program… (N03).
**Guidelines and processes for patient involvement in assessment**		
As a facilitator	NA	NA
As a hindrance	Don’t know the best way to collect that information there is no guidance (DK01). Are you targeting different ethnic groups….How many [patients] are you going to require….these are major issues that need to be sorted out (DK01)? How will they [patients] enter onto the ePortfolio system? Would they receive some sort of token link for a one-time assessment? So, I think that logistics might be challenging…. These guidelines are things that don’t necessarily exist at the moment (Y01). We don’t have a specific process where we can ask for feedback evals (Y02). Lack of knowledge and guidance on how to analyze the data….Like what do I do with it (DK02). I’m not going to bother doing it if it’s not mandatory by the Royal College (DK02). It’s not mandatory…and frankly it is a lot of work to involve the patient so some programs won’t do it (Y02).	I don’t think there’s any way for patients to do assessment because they obviously can’t log on to Mainport through the Royal College (N01). No clear goal and vision of what they’re going to do so if I don’t know how they’re [patients] going to participate, and contribute to the education, how am I going to approach them (N02). You can’t ask just one patient in the day. How many do I ask? How many assessments are needed to know that the information is pertinent (N03)? We don’t know how to involve patients. We have no skills in knowing how to involve patients. It would be from a ground up operation and we would definitely need support and guidance (N04).
**Faculty members’ and residents’ perceptions**		
As a facilitator	It’s good…I think we cannot just ignore it (DK03). It gives you a different view of the resident…you actually get a better sense from the patient (DK04). It [patient assessment] makes the resident better (Y01). They [patients] could give a lot more insight, add insights (Y03). Everyone has different perspectives, everyone sees a patient encounter through a different lens, so that’s the value of having multiples lenses….I think having, you know patients and families involved in some ways is the ultimate lens (Y04).	NA
As a hindrance	There’s still a bias with patients…all those kind of things you don’t want to see (Y02). There will always be a few [residents] who disregard with it because it’s patients and they can’t be trusted…but it’s convincing them that there is significant importance in it and value (Y04).	Yeah no we have never surveyed the patients. The worry is about bias….too many patients at either extremes (N02). We haven’t been sensitized to that [patient involvement] really… (N03). There’s resistance from the faculty. There’s a sort of disengagement just to transition to competence by design. I preach, I preach, but my church is empty and I try to reach them, but those that come to church, it’s always the same people. …if we’re heading in that direction [patient involvement in assessment], the university department head and the hospital department head need to be allies in it, and this in addition to the program director (N03). People [faculty and residents] are not super keen on it and there would be resistance from both the faculty and the residents to gather it…we cuddle our residents and any patient feedback…that would be a huge leap and stretch to start engaging with the patients in a more meaningful way in assessment cause we don’t even get assessments from other healthcare providers right now and we work in a multidisciplinary environment (N04).
**Staffing and time**		
As a facilitator	The way we will remember to give them [patients] the form to fill is by giving it to the clerk, to the reception clerk…to provide to the patient to fill (DK01). If you have someone, a good program administrator that can track the flow of work and ask people to do it, it will be done (DK02). The residents can also go and you know distribute those forms to, to people in the clinic (DK02). Give the onus to the resident to hand those forms over…giving them the responsibility and it wouldn’t be an increased workload on nurses or us (DK04). We could even ask out nurses to hand them [patient assessment questionnaires] and give them [patients] an envelope so they could seal and hand them back before they leave (DK04).	NA
As a hindrance	Someone has to engage patients to explain to them why they’re doing that, collate all this information, then sit down with the residents and discuss it…this means less academic productivity and less clinical productivity (DK01). People [faculty] don’t have time to do that…there’s no time for teaching sometimes (DK03). Frankly it is a lot of work to involve the patient (Y02).	Having somebody actually gather that feedback would be at this point probably not possible because…cause it’s a time thing (N01). Cause it takes time and, uh, staff resources and I, I don’t want to sound cliché but that’s one major barrier (N02). In my opinion, the new curriculum doesn’t demand that we gather this type of data and to be honest with you, there are other things we need to address and there is a hundred other things we need to do and we need to focus our resources and time on those things instead. There are so many required assessments and there would be faculty fatigue in terms of collecting the patient data (N04).
**Availability and existence of patient assessment tools**		
As a facilitator	I think that the 360 assessment format and tools would be good in the sense that we usually pick out very specific things that are relevant to the patient interaction so I definitely think that would be the way to go (Y01). Involve patients in existing 360 assessment tools and systems (Y03). We’re hoping to expand the 360 that we’re doing right now (Y04).	NA
As a hindrance	No reliable tools. There has to be some reliability to the assessment tool. There has to be some validity too (DK01). In my program we don’t have tools for patient assessment (DK02). It would be nice if we could develop other tools so they were standardized obviously for these assessments…these are things that don’t necessarily exist at the moment and can’t do (Y01).	No patient tools exist for our EPAs, you know, that can be incorporated into the Mainport (N01). Don’t know what to use. What are the tools that we can use (N02)? …if a tool existed that we could adopt… there’s like a multiplicity of tools because everyone in their programs, in their university is developing something and we don’t have access to them. …we’re not able to [develop tools]. We don’t have the resources to develop these things (N03).
**Type of Entrustable Professional Activities (EPAs)**		
As a facilitator	In each EPA there’s room for it….the direct patient interaction type of EPAs (DK04). In certain EPAs, you know, you would want to have so many assessments from the family…in order to be deemed competent in that EPA (Y01). I think some of the EPAs would definitely lend themselves to that…you know there’s a whole ton, I think that would lend themselves to patient involvement (Y03).	NA
As a hindrance	It’s going to be a bit of a challenge to make sure that we are directly dealing with the specific questions asked in each EPA (DK04). EPAs not patient-oriented at this point…It doesn’t really fall nicely into EPAs….the way the EPAs have been kind of developed and written out there just potentially is no place for it (Y04).	The EPAs that we’ve been given do not include patients in the assessment (N01). There’s nothing in our EPAs that required patient involvement (N02). When we are mapping out the EPAs and we are tying them to learner experiences, there is no place for patients right now. I don’t see there is much place for patient-specific feedback (N04).
**Type of patient interactions in program**		
As a facilitator	If I’m going to have a patient fill out a form to assess my residents, I’d rather have that longitudinal relationship (DK02). There’s a longitudinal clinic…I mean they [patients] could easily do that in that situation (DK04). We try to match up the residents with a family that they follow over time…. This would be good for families assessing (Y01).	NA
As a hindrance	They may see the person [patient] in the clinic but not see the person on the floor or they be involved in the patient’s care through the emergency department while they’re on-call and never see the patient again…problematic to get reliable feedback (DK01).	We provide indirect or short patient care like initial consults (N01). The question is how are we going to do this? Because currently we have nurses in the operating room, on the ward, and in the outpatient clinic that assess the residents, in addition to the clerks of the outpatient [program specialty] clinic. But the interaction [of the resident] with the patient in the outpatient [program specialty] clinic isn’t very long. Will the resident choose to give their assessment just to patients they clicked with and it went very well (N03)? If we look at most [program specialists], it’s mostly day surgeries. You know, the patients they don’t stay that long at the hospital (N03). The relationship we get with the patient is brief and intense and most the time they don’t remember who we are. We don’t have an opportunity to build a trust opportunity and there is really no way we could get our faculty to get onboard with patient assessment because of this (N04). They [patients] don’t have a face or name relationship with us (N04).
**Support from healthcare organizations**		
As a facilitator	Involving our [hospital] administration....so they feel we are respecting the patient’s privacy…and that we’re not asking for anything that is going to violate privacy (DK04). It’s the family-centred care in the hospital…we cannot just separate the parents from we do (DK03). In pediatric hospitals whereby we’re probably more likely to buy in to the concept of involving patients and families because of family-centred care (Y03). I know over here, you know, patient advisory groups and family advisory groups and things like that it would help in terms of recruiting patients (Y03). I’m told there’s a pool of patients ready to this work. They [hospital] have an infrastructure for contacting and indexing them according to what they have….They have all the contact information (Y02).	NA
As a hindrance	So you know, I don’t know how the hospital would react or feel about this. They have no resources to help us…this is especially an issue for them on how the data will be anonymized (DK01).	Some institutions themselves just happen to have some resources available to them, say for example there may already be sort of patient advisory groups and things like that so maybe pre-existing pools of patient…to engage them in different ways in assessment….we don’t have that (N02).

*Note*.**Y** beside the participant identification number indicates that the interviewee’s program will be involving patients in resident assessment. **DK** beside the participant identification number indicates that the interviewee’s program does not know if it will be involving patients in resident assessment. **N** beside the participant identification number indicates that the interviewee’s program does not involve patients in resident assessment.
